# Influence of age, severity of infection, and co-infection on the duration of respiratory syncytial virus (RSV) shedding

**DOI:** 10.1017/S0950268814001393

**Published:** 2014-06-05

**Authors:** P. K. MUNYWOKI, D. C. KOECH, C. N. AGOTI, N. KIBIRIGE, J. KIPKOECH, P. A. CANE, G. F. MEDLEY, D. J. NOKES

**Affiliations:** 1 KEMRI – Wellcome Trust Research Programme, Centre for Geographic Medicine Research – Coast, Kilifi, Kenya; 2 Public Health England, Salisbury, UK; 3 School of Life Sciences and WIDER, Gibbet Hill Campus, The University of Warwick, Coventry, UK

**Keywords:** Developing countries, Kenya, respiratory syncytial virus, shedding duration, transmission dynamics

## Abstract

RSV is the most important viral cause of pneumonia and bronchiolitis in children worldwide and has been associated with significant disease burden. With the renewed interest in RSV vaccines, we provide realistic estimates on duration, and influencing factors on RSV shedding which are required to better understand the impact of vaccination on the virus transmission dynamics. The data arise from a prospective study of 47 households (493 individuals) in rural Kenya, followed through a 6-month period of an RSV seasonal outbreak. Deep nasopharyngeal swabs were collected twice each week from all household members, irrespective of symptoms, and tested for RSV by multiplex PCR. The RSV G gene was sequenced. A total of 205 RSV infection episodes were detected in 179 individuals from 40 different households. The infection data were interval censored and assuming a random event time between observations, the average duration of virus shedding was 11·2 (95% confidence interval 10·1–12·3) days. The shedding durations were longer than previous estimates (3·9–7·4 days) based on immunofluorescence antigen detection or viral culture, and were shown to be strongly associated with age, severity of infection, and revealed potential interaction with other respiratory viruses. These findings are key to our understanding of the spread of this important virus and are relevant in the design of control programmes.

## INTRODUCTION

Respiratory syncytial virus (RSV) is a major viral cause of lower respiratory tract infection in children worldwide [1] with the key risk group being young infants [2]. No vaccine is currently available for this age group. Development of alternative control strategies depends on the mechanisms of transmission, which are intrinsically related to viral shedding [3, 4]. Detailed data on shedding in individuals in relation to age, infecting subtype (groups A or B), infection severity, and gender would help in identifying the source of infant infection. Such data from the natural setting unaffected by sampling bias are limited and absent in resource-poor settings. Additionally, this study assesses the impact of the presence of other respiratory viruses, prior to or concomitant with RSV, on RSV infection duration. Any interaction might be mediated through direct interference or host immune and physiological responses. These possibilities have received very little attention in the literature.

Previous studies on RSV shedding have been mainly in the hospital setting limiting the generalizability of the results [3, 5, 6]. Hospital studies are biased to young children with severe RSV disease and fail to precisely establish the start and, often, the end of shedding, particularly when symptoms do not coincide with virus shedding which is common for RSV [6]. Community-based studies are likely to provide a more complete representation of the RSV shedding patterns. Such studies require frequent nasopharyngeal swabbing regardless of symptoms and use of sensitive molecular techniques for viral testing in order to minimize the likelihood of missing infection episodes especially in older age groups.

The current prospective study utilizes the above approach, with intensive sampling (every 3–4 days), molecular testing, and follow-up of individuals of all ages for one complete RSV season in a rural Kenyan community [7]. This provides for more realistic estimates on duration of and influencing factors on RSV shedding which are required in designing RSV prevention strategies and to better understand the impact of vaccination on RSV transmission dynamics.

## MATERIALS AND METHODS

The study was undertaken in rural coastal Kenya within the Kilifi Health and Demographic Surveillance System (KHDSS) [8]. The study methods have been described elsewhere [7]. Briefly, a prospective cohort study was undertaken with a recruitment target of 50 RSV-naive infants and their household members. A household was defined as a group of individuals living in the same compound and with common cooking arrangements. Households were eligible if they contained a child born after the end of the 2008–2009 RSV epidemic, and one or more older siblings (aged <13 years). Sampling visits were timed to begin and end coincident with the start and finish of the expected RSV season of 2009–2010 [7]. Trained field assistants made twice weekly household visits, collecting deep nasopharyngeal swabs (NPS) irrespective of symptoms and recording clinical illness data from all participants.

### Study procedures

NPS were collected and tested for RSV (groups A and B) and other respiratory viruses [adenoviruses, rhinoviruses and human coronaviruses (NL63, 229E, OC43)] using real-time multiplex PCR as described previously [7]. In order to establish the genetic similarity of the RSV strains in suspected repeat infections, the ectodomains of the RSV attachment (G) protein gene were sequenced and analysed phylogenetically [9].

### Ethics

The authors assert that all procedures contributing to this work comply with the ethical standards of the relevant national and institutional committees on human experimentation and with the Helsinki Declaration of 1975, as revised in 2008.

### Statistical analysis

Data were analysed using Stata version 11.2 (StataCorp, USA). The infection data were interval-censored and three possible durations of viral shedding were estimated, i.e. minimum, midpoint and maximum, as described in the Supplementary online material and shown in Figure S1. An RSV infection episode was defined as the period within which an individual provided specimens which were PCR positive for the same infecting RSV group with no more than 14 days separating any two positive samples. Episodes where the first sample was positive for both RSV groups A and B counted as one infection episode. Episodes where no samples were collected for >7 days before or after the infection episode were considered left- or right-censored, respectively. Symptomatic infection was defined as the presence of one or more of the following symptoms: cough, nasal discharge/blocked nose, or difficulty in breathing at any time during the infection episode. Co-infection was assigned when within the RSV episode any sample was PCR positive for another virus, i.e. coronavirus, rhinovirus, or adenovirus. Presence of these viruses in the samples collected in the period of 14 days prior to start of RSV episodes was also defined. Household outbreak was defined as a period within which more than one individual episodes occurred in members of the same household without an interval of ⩾14 days in which a PCR-positive specimen was absent from the household. The proportion of the household members infected during household outbreaks measured the intensity of the outbreak.

Cox proportional hazards models were used to identify factors influencing the rate of loss of virus detection (hereafter referred as the recovery rate). The effect of left-censoring was accounted for in the multivariate model by including a dummy variable or by excluding the left-censored episodes.

## RESULTS

### Baseline characteristics of RSV-infected individuals

Of the 493 individuals in the 47 households followed, 179 (36·3%) had at least one RSV infection from 40 (85·1%) different households. The median age (interquartile range; IQR) at the start of the first observed infection was 6·5 (IQR 2·4–14·5) years, and females numbered 96 (53·6%) (Table 1).

**Table 1.** Baseline characteristics of the 179 study participants and the associated RSV infection episodes

Covariates	Categories	Persons^a^ (*N* = 493)		Episodes^b^ (*N* = 205)	
		*n*	%	*n*	%
Age in years, at start of sampling					
	<1 yr	55	11·2	37	18·1
	1–<5 yr	82	16·6	49	23·9
	5–<15 yr	165	33·5	74	36·1
	15–<40 yr	147	29·8	36	17·6
	⩾40 yr	44	8·9	9	4·4
Relations^c^	Study infant	47	9·5	34	16·6
	Sibling	164	33·3	79	38·5
	Cousin	124	25·2	55	26·8
	Mother	46	9·3	14	6·8
	Father	33	6·7	6	2·9
	Other	79	16·0	17	8·3
Gender	Female	272	55·2	116	56·6
	Male	221	44·8	89	43·4
RSV subtype	Group A	—	—	81	39·5
	Group B	—	—	110	53·7
	Groups A and B	—	—	14	6·8
Order of infection	First	—	—	179	87·3
	Second	—	—	26	12·7
Symptomatic, i.e. with ARI	No	—	—	87	42·4
	Yes	—	—	118	57·6
Presence of co-infection	No	—	—	119	58·1
	Yes	—	—	86	42·0
During household outbreak	No	—	—	42	20·5
	Yes	—	—	163	79·5
Outcome					
Duration^d^	<7 days	—	—	74	36·1
	7–<14 days	—	—	75	36·6
	15–<21 days	—	—	32	15·6
	⩾21 days	—	—	24	11·7

RSV, Respiratory syncytial virus; ARI, acute respiratory illness.

a Number of individuals in the parent study.

b RSV infection episodes.

c The relationship of household members with the study infant.

d Duration of RSV shedding episodes.

### RSV infection episodes

**Fig. 1.**
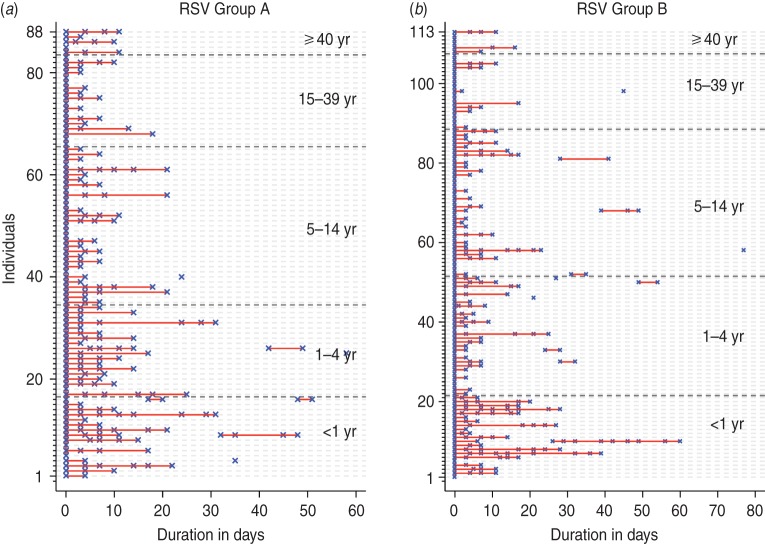
Respiratory syncytial virus (RSV) group A (*a*) and group B (*b*) episodes ordered by age at infection. RSV-positive samples are marked by a blue ‘ × ’ while the red line links PCR-positive samples from the same infection episode.

**Fig. 2.**
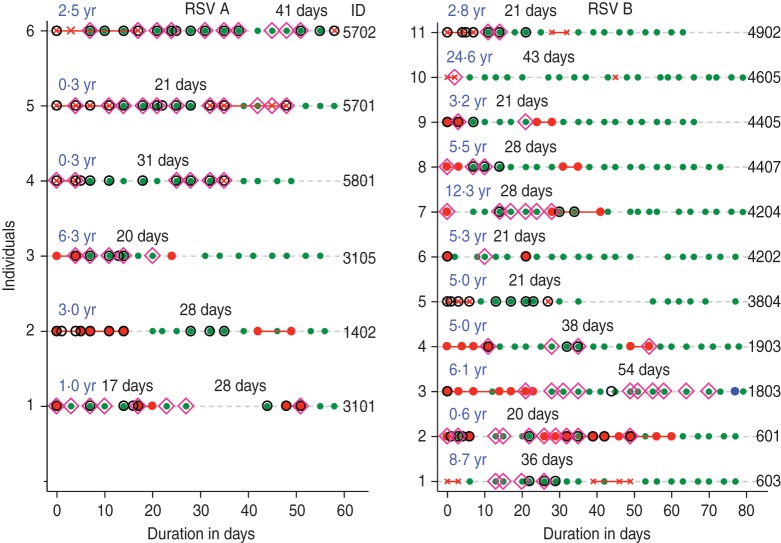
Infection episodes for 17 individuals with suspected repeat infection with the same respiratory syncytial virus (RSV) group showing RSV G gene variability. Green solid circles indicate samples were collected but RSV negative; red crosses (×) indicate RSV-positive samples but not sequenced; solid red or blue circles show RSV-positive samples successfully sequenced. If red is in both episodes then the variants in each episode were identical and if one episode is red and the other blue then they were non-identical. Open pink diamonds (◇) represent detection of other viruses; open black circles (○) show when the individual had respiratory symptoms. Age (in years) at the start of the first episode is in blue, with the corresponding interval between episodes (in days) in grey. ID, Unique identifier of the individual.

A total of 205 infection episodes were observed with 155 individuals experiencing one episode, 22 with two episodes and two individuals experiencing three episodes (Fig. 1). RSV group A was associated with 88 infection episodes, RSV group B with 113 while seven episodes were co-infections. There were 177 (86·3%) fully observed episodes while 11 and 15 infection episodes were left- and right-censored, respectively, and two episodes were both left- and right-censored. Of the 24 individuals with two or more episodes (suspected repeat infections), 17 (70·8%) were infected with the same RSV group and otherwise all except one group A infection followed group B. The mean age at the first infection for individuals with RSV group A and group B was 2·3 and 7·2 years, respectively. The duration between the episodes ranged from 17 to 54 days with median of 28 days. For the 17 homologous infections, sequencing of the RSV G gene was successful in 10 (59%) of the 18 possible pairs of samples (one individual had three suspected RSV episodes). The failure to sequence was mainly in samples with a PCR cycle threshold (C_t_) value of > 28·0, an indicator of low viral load. Only one of the successfully sequenced paired samples showed nucleotide differences: 13 nucleotide differences associated with three non-synonymous changes and a change in the stop codon position. The episodes in this individual (ID no. 1803 in Fig. 2) occurred 54 days apart. For the purposes of estimation of the shedding duration, all the episodes were considered distinct.

### Duration of RSV shedding

**Fig. 3.**
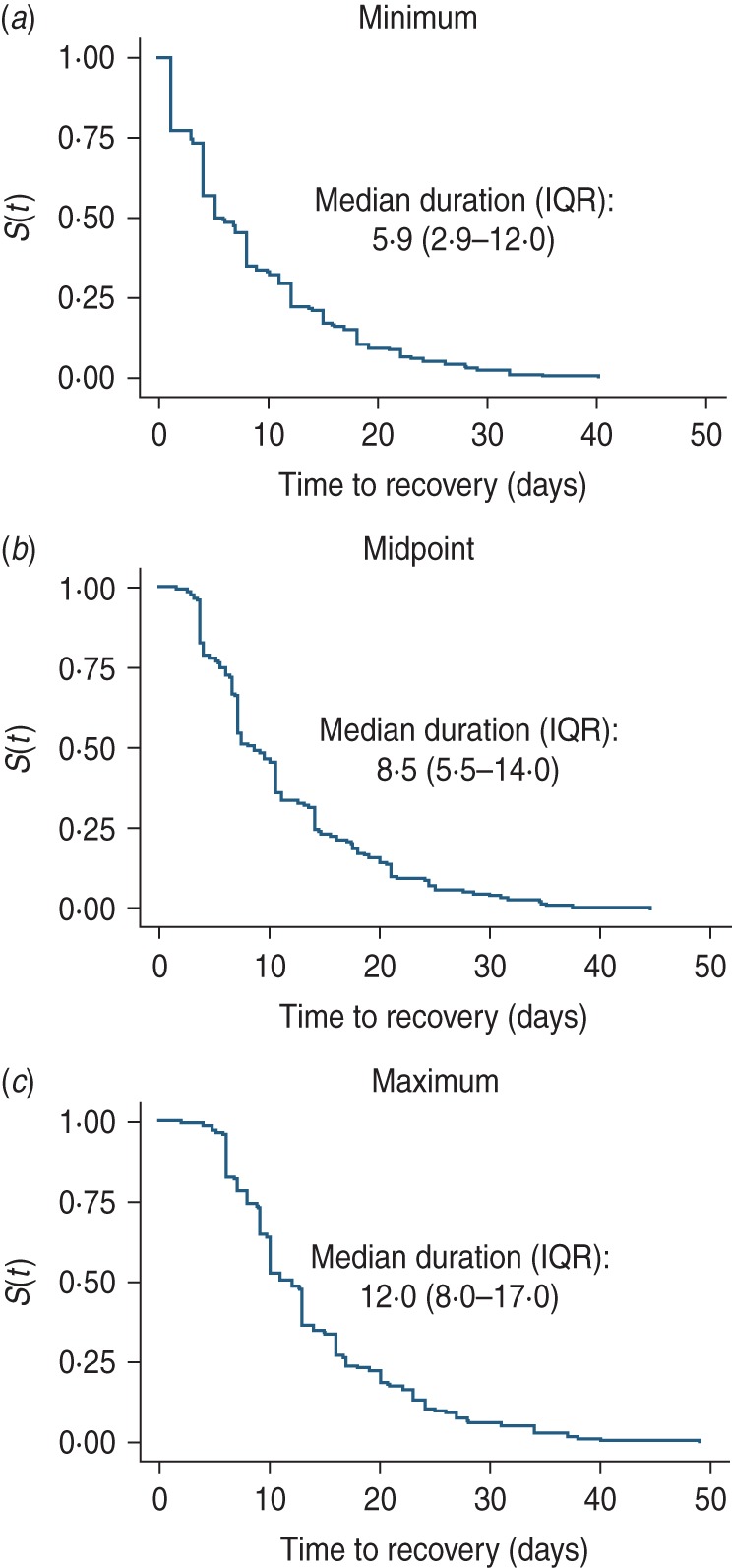
Kaplan–Meier plots showing the (*a*) minimum, (*b*) midpoint and (*c*) maximum estimates of median duration of respiratory syncytial virus shedding. IQR, Interquartile range.

From the 205 infection episodes, the mean duration of shedding based on minimum, midpoint and maximum estimates were 8·6 [95% confidence interval (CI) 7·5–9·7], 11·2 (95% CI 10·1–12·3) and 14·0 (95% CI 12·8–15·2) days, respectively, for all RSV episodes (Fig. 3). The corresponding mean durations of shedding for the ‘fully observed’ episodes were 8·2 (95% CI 7·1–9·4), 10·9 (95% CI 9·8–12·1) and 13·6 (95% CI 12·4–14·8) days, respectively (Table 2). Twenty-four individuals shed RSV for ⩾21 days, and of these 10 (41·7%) were aged <1 year, six (25·0%) were aged 1–4 years, and eight (29·2%) aged 5–17 years. Twenty-two (91·7%) of these infection episodes were symptomatic throughout or at some time point during the shedding. The prolonged shedders contributed 647·5 days of RSV shedding which was 29·7% of the cumulative shedding duration for all episodes based on the midpoint estimation.

**Table 2.** Sampling intervals, age at infection and estimated shedding duration by the censoring type

Characteristic	All^a^ (*n* = 205)		Fully^b^ (*n* = 177)		Left^c^ (*n* = 11)		Right^d^ (*n* = 15)		Both^e^ (*n* = 2)	
Sampling intervals^f^	Mean	95% CI	Mean	95% CI	Mean	95% CI	Mean	95% CI	Mean	95% CI
Before	4·2	3·8–4·5	3·7	3·6–3·9	9·6	8·6–10·5	3·4	2·8–4·0	18·50	—
During	3·5	3·4–3·6	3·5	3·4–3·6	3·8	2·8–4·9	4·1	3·1–5·0	—	—
After	5·2	4·2–6·3	3·7	3·5–3·8	3·4	2·4–4·3	24·1	13·3–34·9	11·0	—
Age, years^g^	11·1	9·3–13·0	10·4	8·6–12·3	15·1	5·0–25·1	15·6	6·5–24·6	16·9	—
Shedding duration^h^	10·6	9·6–11·7	10·9	9·7–12·1	7·4	4·5–10·2	8·9	6·7–11·0	14·8	—

CI, Confidence interval; RSV, respiratory syncytial virus.

a All RSV infection episodes; ^b^ fully observed episodes; ^c^ left-censored episodes only; ^d^ right-censored episodes only; ^e^ episodes with both left and right censoring; ^f^ intervals between nasopharyngeal swab collection before, during, and after the RSV infection episode in days; ^g^ age (in years) at the start of the episode; ^h^ shedding duration (in days) based on the midpoint estimation.

**Fig. 4.**
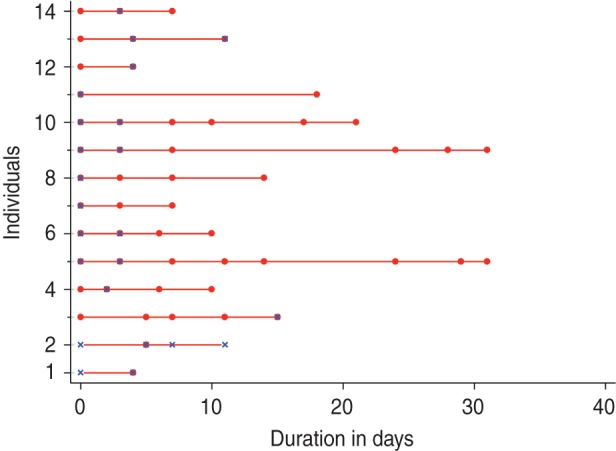
Respiratory syncytial virus (RSV) shedding in episodes associated with co-detection of RSV groups A and B. The solid red circle (●) and blue marker (×) represents RSV groups A and B, respectively. The red line links PCR-positive samples denoting the RSV episode.

In 14 infected individuals, one or more samples were identified to contain both RSV groups A and B. The timing of these co-detections is shown in Figure 4. In most (12/14) episodes, RSV group A was shed for longer duration relative to group B.

### Factors influencing the rates of recovery from RSV infection

The hazard ratios (HRs) for the various factors from univariate Cox regression were similar for minimum, midpoint and maximum estimates data (Supplementary Table S1). The midpoint data were taken forward for the multivariate analysis and the final model is reported in Table 3. The results were similar without and with inclusion of the left-censored RSV episodes (Table 3 and Supplementary Table S2, respectively). The proportionality assumption in the Cox regression model was not violated based on the test of the Schoenfeld residuals (Supplementary Table S3).

**Table 3.** Final multivariate Cox regression model^*a*^: factors influencing the rates of recovery from RSV infection in rural Kenya

Factors	Categories	Reference	HR	95% CI	*P* value
Age group, years					
	1–4 yr	<1 year	1·98	1·30–3·02	0·002
	5–14 yr		1·82	1·16–2·87	0·01
	⩾15 yr		1·97	1·11–3·51	0·021
Symptomatic		Asymptomatic	0·56	0·40–0·79	0·001
Detection of other^b^ viruses before and during RSV episode	Before^c^	No other viruses	1·56	1·02–2·39	0·041
	During		0·48	0·32–0·73	<0·001
Other household members infected (%)	⩾33%–66%	<33%	0·59	0·40–0·87	0·008
	>66%		0·51	0·35–0·74	<0·001
RSV group B		A	1·07	0·83–1·39	0·588
Male gender		Female	0·97	0·77–1·23	0·804
Second RSV episode		First	0·91	0·53–1·56	0·73

RSV, Respiratory syncytial virus; HR, hazard ratio; CI, confidence interval.

a Left-censored episodes excluded.

b Other viruses were adenoviruses, rhinoviruses and coronaviruses.

c Detection of other respiratory viruses during the 14 days prior to the start of RSV episode only.

The rate of recovery from RSV infection was age-dependent. The adjusted HR (aHR) were 1·98 (95% 1·30–3·02), 1·82 (95% CI 1·16–2·87), 2·10 (95% CI 1·20–3·66) and 1·31 (95% CI 0·36–4·81) in the 1–4, 5–14, 15–39 and ⩾40 years age groups, respectively, relative to infants (<1 year). The rate of recovery was lower by 44% in symptomatic infections relative to asymptomatic infections (aHR 0·56, 95% CI 0·40–0·79). The presence of one or more additional viruses (rhinovirus, coronavirus, adenovirus) was detected in 86 RSV infection episodes. The rate of RSV recovery was lower (i.e. shedding duration increased) by 65% in episodes with co-infection compared to those without (aHR 0·35, 95% CI 0·23–0·51), with a similar result for each virus individually. Detection of infection with any one or more of rhinovirus, adenovirus or coronavirus, in the 2 weeks preceding the start of RSV infection, but not during the RSV episode itself, was associated with a 56% increase in the rate of recovery (i.e. reduced shedding duration) from the RSV infection (aHR 1·56, 95% CI 1·02–2·39). In contrast, RSV episodes associated with detection of other viruses in the 2 weeks prior to and also during the RSV infection were associated with a 52% decrease in the rate of recovery relative to those with no other virus prior to and during the RSV episode (aHR 0·48, 95% CI 0·32–0·73). The rate of recovery of RSV episodes associated with spread in the household (outbreaks) was 42% lower than the single household episodes (aHR 0·58, 95% CI 0·43–0·78). A variable denoting the proportion of individuals infected during the household outbreak improved the model fit and was used in the multivariate analyses (likelihood ratio test *P* = 0·0229).

The rate of recovery did not differ significantly by gender and infecting RSV group (aHR 0·97, 95% CI 0·77–1·23 and aHR 1·07, 95% CI 0·83–1·39, respectively). Recovery rate was similar in suspected repeat infections compared to the first observed episodes (aHR 0·91, 95% CI 0·53–1·56).

## DISCUSSION

We observed 205 infections with RSV during one epidemic with a most realistic estimate of 11·2 (95% CI 10·1–12·3) days of shedding. The most conservative and least conservative estimates were 8·6 days and 14·0 days, respectively. The duration of shedding based on the most realistic estimate decreased with age: 18 days in infants and 9 days in adults (aged ⩾15 years). Symptomatic infections on average had longer virus shedding of 13·5 days compared to 7·8 days in asymptomatic episodes. The presented average durations of virus shedding are longer than published estimates of 6·7 days [4], 3·4–7·4 days [10], 3·9 days [11], and 4·5 days [12] which could be attributed to differences in study methods. The present study was informed by critical review of the previous studies and it incorporated frequent sampling regardless of symptoms and screening by highly sensitive PCR methods. The specimen collection procedure was acceptable, recording a good compliance across all ages [7, 13]. The use of the sensitive viral detection method (PCR) results is likely to result in longer estimates of shedding.

A community study nested within a birth cohort in coastal Kenya targeting symptomatic RSV infections by Okiro and colleagues reported a mean duration of shedding of 4·5 days [12]. Our corresponding estimate in symptomatic cases was 13·5 days. In a subset of the children whose start of symptoms could be established from the clinic records, the Okiro *et al.* study reported a longer duration of 7·7 days [12]. Given that RSV shedding has been reported to start before illness [6], the actual duration in the symptomatic children would have been an underestimate and our estimate of 13·5 days is likely to be more accurate. A Rochester family study, in the USA, with similar design as the present study (collecting samples every 3–4 days regardless of symptoms) reported lower estimates of duration of shedding of 3·4–7·4 days [10]. The Okiro *et al*. and Rochester study used the immunofluorescent antibody test (IFAT) and culture, respectively, which are less sensitive methods [14]. As a counter argument, it is not known to what degree PCR positivity equates with shedding of viable and infectious virus. Thus, while the molecular methods might be more sensitive, the resultant increase in duration of shedding over more traditional methods such as culture (which directly measures viral infectivity) may not necessarily translate to increased period of infectivity. Further work relating virus infectiousness and detectability is warranted. In the Okiro *et al*. study, sampling started when participants were symptomatic and stopped at the first negative follow-up sample. The present study revealed instances where negative samples arose within RSV infection episodes. Even though this observation raises, again, questions on the relationship between infectivity and shedding duration, accounting for periods of RSV-negative samples would still result to longer shedding duration compared to previous estimates. Alternative estimation of the shedding patterns by calculating the area under the C_t_ (viral load) curve would have some additional advantages and will be explored in future.

Prolonged shedders of > 3 weeks' duration have been reported [6, 10]. In the present study, 24 (13·4%) episodes in the 179 infected individuals involved shedding RSV for >3 weeks. Most (22, 91·7%) of these episodes were symptomatic, and occurred in young children (median age 14·7 months). Individuals with compromised immunity have been known to shed RSV for longer [15] but participants in the current study were not tested for HIV. The HIV prevalence in women and men aged 15–49 years in coastal Kenya was 4·2% according to the Kenyan Demographic and Health Survey of 2008/2009 [16]. In settings where HIV prevalence is high, the effect of the poor viral clearance might influence the temporal epidemiology as was observed in South Africa [15]. There was no obvious malnourished participant in the study cohort based on mid-upper arm circumference measurements.

More than two RSV episodes were observed in 24 (13·4%) individuals. On average, the episodes were 4 weeks apart. Most (70·8%) of the suspected repeat infections were with homologous RSV group. Sequencing of the most variable region of the RSV genome, the ectodomain of the G gene, did not greatly assist in resolving the infection episodes as most (9/10) had identical sequences. It is, thus, not clear whether the two phases of RSV shedding were repeat infections with the same variant or were persistent infection with periods of low viral load that was undetectable by the methods used. Using post-mortem lung tissue from infants, RSV RNA has been detected even in children dying during inter-epidemic periods suggesting the persistence of RSV in the lungs of these infants [17]. Similar observations have been made in experimental infection with RSV [18] and measles viruses [19]. The lack of variability in the virus identified in the two phases of infections suggests virus mutation might not be the primary mechanism for virus persistence or re-infection. Regardless of whether it is re-infection within a short period or persistence, the observation represents an interesting phenomenon of RSV which has potential importance on our existing view of acute RSV infection, the development of immunity and effects on viral transmission.

The age of the individual, infection severity, detection of other viruses before and during the RSV infection, and presence of concurrent RSV infections in the same household were all associated with virus shedding. The rate of recovery increased with increasing age, with individuals in 1–4 years, 5–15 years and ⩾15 years age groups recovering 1·98, 1·82 and 1·97 times faster than the infants, respectively. The Rochester family study reported similar findings where longer shedding was observed for children aged <2 years compared to those aged 2–16 years (9 *vs.* 4 days). The Okiro *et al.* study did not find any association with age but found that children with previous RSV infection (using the assumption that those aged >3 years were by default experiencing a repeat infection) had 1·37 times faster rate of recovery compared to those without a history of infection [12]. In the present study, a subsequent RSV infection during the same RSV season was not significantly associated with reduced shedding duration, but such infections were few (*n* = 24). The current study is for one epidemic only, so age must act as a proxy of exposure to RSV in earlier epidemics. Prolonged shedding enhances the possibility of person-to-person transmission and makes young children a potential source of community spread of infection, both of which have important implications in the control and prevention of RSV infection.

A study involving 23 hospitalized children (aged <2 years) with sampling extended beyond discharge reported an association of duration of shedding and symptom severity [4]. Children with lower respiratory tract infection shed for longer than those with upper respiratory tract infection (8·4 *vs*. 1·4 days). Duration of shedding may be related to severity of disease but evidence is controversial on the link between disease severity and viral load [5, 20–22].

An interaction between detection of other viruses (i.e. coronavirus, rhinovirus, adenovirus) in the nasopharynx and rate of recovery from RSV infection was observed. Recovery from a viral infection just prior to RSV might have led to up-regulation of innate viral immunity or non-specific cross-reactivity that reduced subsequent RSV shedding. Presence of co-infections might be a marker of low immunity associated with poor viral clearance. RSV episodes with concurrent spread in the household were associated with increased recovery rate. However, any conclusions other than association cannot be made since the extended duration of shedding increases the risk of both co-infection with other viruses and multiple RSV infections in the household. A different estimation framework is required to untangle these results.

The problems of ascertainment and analysis (i.e. censoring and test sensitivity) are not completely eliminated by the careful study design, but did not seem to affect the association of the examined factors with virus shedding (Supplementary Table S1). Short RSV infections occurring between specimen collections particularly in older individuals might have been missed but this is likely to be at random and bias hazard ratios towards null.

In conclusion, this study defines RSV shedding patterns in the natural setting with significant potential for improved understanding of the spread of this important virus and with relevance the design of control programmes.

## Supplementary material

For supplementary material accompanying this paper visit http://dx.doi.org/10.1017/S0950268814001393. click here to view supplementary material

## References

[1] Nair H, Global burden of acute lower respiratory infections due to respiratory syncytial virus in young children: a systematic review and meta-analysis. Lancet 2010; 375: 1545–1555. 20399493 10.1016/S0140-6736(10)60206-1 PMC2864404

[2] Glezen WP, Risk of primary infection and reinfection with respiratory syncytial virus. American Journal of Diseases of Children 1986; 140: 543–546. 3706232 10.1001/archpedi.1986.02140200053026

[3] Waris M, Shedding of infectious virus and virus antigen during acute infection with respiratory syncytial virus. Journal of Medical Virology 1992; 38: 111–116. 1460457 10.1002/jmv.1890380208

[4] Hall CB, Douglas RG Jr., Geiman JM. Respiratory syncytial virus infections in infants: quantitation and duration of shedding. Journal of Pediatrics 1976; 89: 11–15. 180274 10.1016/s0022-3476(76)80918-3

[5] Hall CB, Douglas RG Jr., Geiman JM. Quantitative shedding patterns of respiratory syncytial virus in infants. Journal of Infectious Diseases 1975; 132: 151–156. 808581 10.1093/infdis/132.2.151

[6] Frank AL, Patterns of shedding of myxoviruses and paramyxoviruses in children. Journal of Infectious Diseases 1981; 144: 433–441. 6273473 10.1093/infdis/144.5.433

[7] Munywoki PK, The source of respiratory syncytial virus infection in infants: a household cohort study in rural Kenya. Journal of Infectious Diseases. Published online: 25 12 2013. doi: 10.1093/infdis/jit828. PMC4017365 24367040

[8] Scott JA, Profile: The Kilifi Health and Demographic Surveillance System (KHDSS). International Journal of Epidemiology 2012; 41: 650–657. 22544844 10.1093/ije/dys062 PMC3396317

[9] Agoti CN, Genetic relatedness of infecting and reinfecting respiratory syncytial virus strains identified in a birth cohort from rural kenya. Journal of Infectious Diseases 2012; 206: 1532–1541. 22966119 10.1093/infdis/jis570 PMC3475639

[10] Hall CB, Respiratory syncytial virus infections within families. New England Journal Medicine 1976; 294: 414–419. 10.1056/NEJM197602192940803 173995

[11] Hall CB, Long CE, Schnabel KC. Respiratory syncytial virus infections in previously healthy working adults. Clinical Infectious Diseases 2001; 33: 792–796. 11512084 10.1086/322657

[12] Okiro EA, Duration of shedding of respiratory syncytial virus in a community study of Kenyan children. BMC Infectious Diseases 2010; 10: 15. 20096106 10.1186/1471-2334-10-15 PMC2822777

[13] Munywoki PK, Improved detection of respiratory viruses in pediatric outpatients with acute respiratory illness by real-time PCR using nasopharyngeal flocked swabs. Journal of Clinical Microbiology 2011; 49: 3365–3367. 21775539 10.1128/JCM.02231-10 PMC3165583

[14] Reis AD, Comparison of direct immunofluorescence, conventional cell culture and polymerase chain reaction techniques for detecting respiratory syncytial virus in nasopharyngeal aspirates from infants. Journal of the São Paulo Institute of Tropical Medicine 2008; 50: 37–40. 10.1590/s0036-46652008000100008 18327485

[15] Madhi SA, Increased burden of respiratory viral associated severe lower respiratory tract infections in children infected with human immunodeficiency virus type-1. Journal of Pediatrics 2000; 137: 78–84. 10891826 10.1067/mpd.2000.105350

[16] Kenya National Bureau of Statistics (KNBS) and ICF Macro. Kenya Demograpghic and Health Survey 2008–09. Calverton, Maryland, USA: KNBS and ICF Macro, 2010.

[17] Cubie HA, Detection of respiratory syncytial virus nucleic acid in archival postmortem tissue from infants. Pediatric Pathology and Laboratory Medicine 1997; 17: 927–938. 9353832

[18] Schwarze J, Latency and persistence of respiratory syncytial virus despite T cell immunity. American Journal of Respiratory Critical Care Medicine 2004; 169: 801–805. 14742302 10.1164/rccm.200308-1203OC

[19] Lin WH, Prolonged persistence of measles virus RNA is characteristic of primary infection dynamics. Proceedings of the National Academy of Sciences USA 2012; 109: 14989–14994. 10.1073/pnas.1211138109 PMC3443140 22872860

[20] DeVincenzo JP, El Saleeby CM, Bush AJ. Respiratory syncytial virus load predicts disease severity in previously healthy infants. Journal of Infectious Diseases 2005; 191: 1861–1868. 15871119 10.1086/430008

[21] Kuypers J, Wright N, Morrow R. Evaluation of quantitative and type-specific real-time RT-PCR assays for detection of respiratory syncytial virus in respiratory specimens from children. Journal of Clinical Virology 2004; 31: 123–129. 15364268 10.1016/j.jcv.2004.03.018 PMC7128826

[22] Devincenzo JP. Natural infection of infants with respiratory syncytial virus subgroups A and B: a study of frequency, disease severity, and viral load. Pediatric Research 2004; 56: 914–917. 15470202 10.1203/01.PDR.0000145255.86117.6A

