# Supplementary material for: Influence of age, severity of infection, and co-infection on the duration of respiratory syncytial virus (RSV) shedding
Source: Epidemiol Infect. 2014 Jun 5;143(4):804–12. doi: 10.1017/S0950268814001393 (PMC4411640; doi:10.1017/S0950268814001393)
Supplement: Supplementary file 1 [file S0950268814001393sup001.docx]

**Epidemiology and Infection**

Influence of age, severity of infection, and co-infection on the duration of respiratory syncytial virus (RSV) shedding

Patrick K. Munywoki^1*^; Dorothy Koech^1^; Charles N. Agoti^1^; Nuru Kibirige^1^; James Kipkoech^1^; Patricia A. Cane^2^; Graham F. Medley^2^; D. James Nokes^1, 2^

Affiliations

1. KEMRI - Wellcome Trust Research Programme, Centre for Geographic Medicine Research – Coast, Hospital Road, P.O. Box 230-80108, Kilifi, Kenya; Tel +254 (0)417 522535; Fax: +254 (0)417 522390
2. School of Life Sciences and WIDER, Gibbet Hill Campus, The University of Warwick, Coventry, CV4 7AL, United Kingdom; Tel: +44 (0)24 7657 4251 Fax: +44 (0)24 7652 2052

*Corresponding Author

Patrick Kiio Munywoki, KEMRI - Wellcome Trust Research Programme, Centre for Geographic Medicine Research – Coast, Hospital Road, P.O. Box 230-80108, Kilifi, Kenya; Tel +254 (0)417 522535; Fax: +254 (0)417 522390; email: [pmunywoki@kemri-wellcome.org](mailto:pmunywoki@kemri-wellcome.org)

**SUPPLEMENTARY MATERIAL**

**Methods**

*Estimation of shedding durations*

If we assume the first day of an infection episode is defined as *i* and the last day as *j*, and the first negative sample day prior to the infection episode defined as *k* and the first negative sample after the last positive sample of an episode defined as *l*, assigning day *k* arbitrarily a value 0 increasing by daily units up to day *l*. Further, assume that *i* and *j* contribute each one full day of shedding and days *k* and *l* contribute 0 days of shedding, then for:

1. Minimum duration: shedding begins on day *i* and ends on day *j* with the shedding duration defined as *j–i+*1 days
2. Midpoint duration: shedding begins mid-way between day *k*+1 and day *i*, and ends mid-way between date *j* and date *l-1*, with the shedding interval defined as *(j+(l-j)/2)-(i-(i-k)/2).* For left and right censored , one half of the average interval between sample collections was subtracted from *i* and added to *j* to define the start and end of shedding, respectively.
3. Maximum duration: shedding begins a day after *k* and ends a day before *l*, hence the shedding duration is defined as *l*-*k*-1. Where observations *k* and *l* were not made (left and right-censored respectively) the average interval between sample collections was subtracted and added to define start and end dates of the shedding, respectively.

Figure S1: Schematic diagram illustrating the timeline for each individual and important time points used in estimating shedding durations. Minimum estimates, j–i+1, Midpoint estimates, (j+(l-j)/2)-(i-(i-k)/2), and maximum estimates, l-k-1

**Results**

*Sampling intervals*

Table 2 shows the distribution of sampling intervals, age at infection and estimated shedding duration by the censoring type. The average interval between sample collections during RSV infection episodes was 3.5 (95% confidence interval, CI; 3.4 – 3.6) days (supplementary Figure S1). The mean intervals prior to and immediately after the infection episodes for the fully observed episodes were not statistically different; 3.7 (95% CI, 3.6 – 3.9) versus 3.7 (95% CI, 3.6 – 3.8) days respectively; t-test P-value=0.4819. There was no association of sample collection hour and RSV detection (Supplementary Figure S2), hence all the shedding duration estimates assumed the samples were collected at the same time of the day.

*Factors influencing the rates of recovery from RSV infection*

The Kaplan-Meier plots showing the association of the various factors and recovery probability arising from midpoint analyses are shown in supplementary Figures S4 and S5.

Table S1: Univariate Cox regression analysis: Factors affecting the rates of RSV recovery

| **Factors** | | | **Baseline** | **Minimum estimates** | | | | **Midpoint estimates** | | | | **Maximum estimates** | | | |
| --- | --- | --- | --- | --- | --- | --- | --- | --- | --- | --- | --- | --- | --- | --- | --- |
|  |  |  |  | HR | LCL | UCL | P-value | HR | LCL | UCL | P-value | HR | LCL | UCL | P-value |
| Age groups | | 1-4y | <1year | 1.89 | 1.17 | 3.06 | 0.009 | 1.91 | 1.18 | 3.09 | 0.008 | 1.78 | 1.10 | 2.86 | 0.018 |
|  |  | 5-14y |  | 2.75 | 1.73 | 4.37 | <0.001 | 2.78 | 1.75 | 4.42 | <0.001 | 2.53 | 1.61 | 3.98 | <0.001 |
|  |  | 15-39y |  | 3.19 | 1.88 | 5.42 | <0.001 | 3.23 | 1.90 | 5.48 | <0.001 | 2.61 | 1.56 | 4.36 | <0.001 |
|  |  | ≥40y |  | 1.80 | 0.77 | 4.20 | 0.173 | 1.92 | 0.83 | 4.49 | 0.13 | 1.85 | 0.80 | 4.29 | 0.152 |
| Relationships | | Siblings* | Study infants | 2.14 | 1.42 | 3.21 | <0.001 | 2.19 | 1.46 | 3.29 | <0.001 | 2.02 | 1.35 | 3.01 | 0.001 |
|  |  | Others |  | 2.46 | 1.47 | 4.11 | 0.001 | 2.70 | 1.61 | 4.51 | <0.001 | 2.57 | 1.54 | 4.28 | <0.001 |
| Symptomatic episode | | | Asymptomatic | 0.48 | 0.35 | 0.65 | <0.001 | 0.45 | 0.33 | 0.61 | <0.001 | 0.47 | 0.34 | 0.64 | <0.001 |
| Presence of other viruses | | | None | 0.45 | 0.33 | 0.61 | <0.001 | 0.43 | 0.32 | 0.59 | <0.001 | 0.47 | 0.35 | 0.64 | <0.001 |
| Other viruses detected | Adenovirus | | None | 0.66 | 0.41 | 1.05 | 0.08 | 0.61 | 0.38 | 0.97 | 0.038 | 0.63 | 0.40 | 1.02 | 0.059 |
|  | Coronavirus | |  | 0.44 | 0.27 | 0.72 | 0.001 | 0.42 | 0.26 | 0.69 | 0.001 | 0.47 | 0.29 | 0.77 | 0.003 |
|  | Rhinovirus | |  | 0.36 | 0.22 | 0.61 | <0.001 | 0.35 | 0.21 | 0.59 | <0.001 | 0.40 | 0.24 | 0.67 | <0.001 |
|  | Mixed | |  | 0.40 | 0.25 | 0.66 | <0.001 | 0.38 | 0.23 | 0.62 | <0.001 | 0.42 | 0.26 | 0.68 | <0.001 |
| Others infected in HH (%) | 33 – <66% | | <33% | 0.59 | 0.42 | 0.83 | 0.003 | 0.61 | 0.43 | 0.86 | 0.005 | 0.68 | 0.48 | 0.95 | 0.024 |
|  | ≥66% | |  | 0.53 | 0.36 | 0.76 | 0.001 | 0.54 | 0.38 | 0.78 | 0.001 | 0.58 | 0.41 | 0.84 | 0.004 |
| Primary cases of HH episodes | | | Non-primary cases | 1.11 | 0.83 | 1.49 | 0.472 | 1.08 | 0.81 | 1.44 | 0.614 | 1.03 | 0.77 | 1.38 | 0.822 |
| Primary cases of HH outbreaks | | | Non-primary cases | 0.69 | 0.48 | 0.99 | 0.043 | 0.68 | 0.47 | 0.97 | 0.033 | 0.69 | 0.48 | 0.99 | 0.044 |
| During HH outbreak | | | No outbreak | 0.51 | 0.35 | 0.73 | <0.001 | 0.49 | 0.34 | 0.71 | <0.001 | 0.52 | 0.36 | 0.74 | <0.001 |
| No. of other viruses | | 1 | None | 0.47 | 0.34 | 0.66 | <0.001 | 0.45 | 0.32 | 0.62 | <0.001 | 0.49 | 0.35 | 0.68 | <0.001 |
|  |  | 2 |  | 0.40 | 0.24 | 0.66 | <0.001 | 0.38 | 0.23 | 0.63 | <0.001 | 0.41 | 0.25 | 0.69 | 0.001 |
|  |  | ≥3 |  | 0.50 | 0.12 | 2.05 | 0.339 | 0.47 | 0.12 | 1.90 | 0.288 | 0.48 | 0.12 | 1.97 | 0.31 |
| Male gender | | | Female | 1.03 | 0.77 | 1.38 | 0.836 | 1.02 | 0.76 | 1.36 | 0.916 | 0.99 | 0.74 | 1.32 | 0.919 |
| RSV group | | Group B | Group A | 1.08 | 0.79 | 1.46 | 0.642 | 1.04 | 0.77 | 1.42 | 0.782 | 1.03 | 0.76 | 1.40 | 0.852 |
|  |  | Both |  | 0.53 | 0.29 | 0.97 | 0.039 | 0.52 | 0.28 | 0.94 | 0.032 | 0.55 | 0.30 | 1.00 | 0.048 |
| Second RSV episode | | | First observed | 1.36 | 0.90 | 2.07 | 0.149 | 1.28 | 0.84 | 1.94 | 0.248 | 1.20 | 0.79 | 1.82 | 0.4 |
| Other viruses detected prior to RSV infection | | | None | 0.85 | 0.64 | 1.14 | 0.283 | 0.86 | 0.65 | 1.15 | 0.32 | 0.90 | 0.67 | 1.20 | 0.463 |
| Viruses detected prior to RSV infection | | Adenovirus | No detections | 0.97 | 0.60 | 1.58 | 0.914 | 1.00 | 0.61 | 1.62 | 0.984 | 1.03 | 0.63 | 1.67 | 0.914 |
|  |  | Coronavirus |  | 1.00 | 0.60 | 1.66 | 0.996 | 1.01 | 0.61 | 1.67 | 0.977 | 1.05 | 0.63 | 1.74 | 0.862 |
|  |  | Rhinovirus |  | 0.86 | 0.58 | 1.27 | 0.445 | 0.87 | 0.58 | 1.28 | 0.472 | 0.91 | 0.61 | 1.35 | 0.635 |
|  |  | Mixed |  | 0.70 | 0.44 | 1.09 | 0.117 | 0.70 | 0.45 | 1.11 | 0.129 | 0.73 | 0.46 | 1.14 | 0.167 |
| No. of viruses detected prior to RSV infection | | 1 | 0 | 0.92 | 0.68 | 1.25 | 0.603 | 0.93 | 0.68 | 1.27 | 0.655 | 0.97 | 0.71 | 1.32 | 0.854 |
|  |  | 2 |  | 0.74 | 0.47 | 1.17 | 0.195 | 0.75 | 0.47 | 1.19 | 0.222 | 0.77 | 0.49 | 1.22 | 0.272 |
|  |  | ≥3 |  | 0.31 | 0.04 | 2.22 | 0.244 | 0.30 | 0.04 | 2.13 | 0.226 | 0.31 | 0.04 | 2.23 | 0.245 |
| Other viruses detected prior to and during RSV episode | | Before only | None | 1.21 | 0.82 | 1.79 | 0.334 | 1.31 | 0.89 | 1.94 | 0.172 | 1.39 | 0.94 | 2.06 | 0.096 |
|  |  | Both |  | 0.49 | 0.34 | 0.70 | <0.001 | 0.48 | 0.33 | 0.69 | <0.001 | 0.53 | 0.37 | 0.76 | 0.001 |
|  |  | During only |  | 0.47 | 0.29 | 0.77 | 0.002 | 0.46 | 0.29 | 0.75 | 0.002 | 0.52 | 0.32 | 0.84 | 0.007 |
| Left censor | | | Not left censor | 1.55 | 0.84 | 2.86 | 0.163 | 2.11 | 1.14 | 3.91 | 0.017 | 2.19 | 1.18 | 4.04 | 0.013 |

Key: HR, hazard ratio; LCL, lower confidence limit; UCL, upper confidence limit

Table S2: Final multivariate Cox regression model^1^: Factors affecting the rates of RSV recovery

| **Factors** | **Categories** | **Baseline** | **HR** | **95% CI** | **P-value** |
| --- | --- | --- | --- | --- | --- |
| Age groups | 1-4y | <1 year | 1.85 | 1.22 – 2.80 | 0.004 |
|  | 5-14y |  | 1.77 | 1.14 – 2.75 | 0.01 |
|  | ≥15y |  | 1.67 | 0.93 – 2.99 | 0.084 |
| Symptomatic | | Asymptomatic | 0.59 | 0.42 – 0.82 | 0.002 |
| Detection of other^2^ viruses before and during RSV episode | Before^3^ | No other viruses | 1.55 | 1.02 – 2.35 | 0.041 |
|  | During |  | 0.50 | 0.35 – 0.70 | <0.001 |
| Other HH members infected (%) | 33 – <66% | <33% | 0.58 | 0.42 – 0.82 | 0.002 |
|  | ≥ 66% |  | 0.57 | 0.41 – 0.80 | 0.001 |
| RSV group B | | Group A | 1.24 | 0.96 – 1.61 | 0.1 |
| Male gender | | Female | 1.00 | 0.78 – 1.27 | 0.992 |
| Second RSV infection | | First | 0.95 | 0.54 – 1.68 | 0.857 |
| Left censored | | Not left censored | 1.88 | 0.76 – 4.65 | 0.172 |

Key: HR, hazard ratio; CI, confidence interval; HH, household; 1, includes left censored episodes; 2, detection of other viruses (rhinoviruses, adenoviruses and coronaviruses) during the RSV episode; 3, detection of other respiratory viruses during the 14 days prior to the start of RSV episode ONLY

Table S3: Test of proportional-hazards assumption using Schoenfeld residuals

| **Factor** | **Categories** | **Rho** | **Chi-square statistic** | **P value** |
| --- | --- | --- | --- | --- |
| Age groups | 1-4y | 0.0028 | 0 | 0.9726 |
|  | 5-14y | 0.01222 | 0.02 | 0.8776 |
|  | 15-39y | -0.01208 | 0.03 | 0.8597 |
| Symptomatic | | 0.01549 | 0.05 | 0.8284 |
| Detection of other viruses before and during RSV episode | Before | -0.00864 | 0.02 | 0.8989 |
|  | During | 0.02406 | 0.12 | 0.7297 |
| Other HH members infected (%) | 33 – <66% | -0.03005 | 0.2 | 0.6517 |
|  | ≥ 66% | 0.02584 | 0.1 | 0.7461 |
| RSV group B | | 0.00053 | 0 | 0.9949 |
| Male gender | | 0.11266 | 1.62 | 0.203 |
| Second RSV infection | | -0.00577 | 0.01 | 0.9223 |
| Global test | |  | 2.52 | 0.9957 |

Key: HH, household

Figure S2: (a) Frequency distribution of the total, RSV group A and RSV group B positive, sample collections by time of collection and (b) percentage of the samples positive for RSV group A, RSV group B or either group by time of sample collection. ‘m’ on the x-axis represents samples with missing time of collection

Figure S3: Distribution of the intervals between NPS collections during RSV negative (a) and PCR positive periods. The red dashed vertical line represents the mean interval; $, in 29 instances the interval between RSV negative samples was more than 30 days

Figure S4: Kaplan-Meier survival function plots of midpoint data for cessation of RSV shedding stratified by age at infection in years (a), RSV groups (b), Gender (c), order of RSV infection episodes (d), presence of acute respiratory symptoms during the RSV episode (e) and number of other co-infecting viruses (f). The legend for each graph is shown in the respective inserted text box.

Figure S5: Kaplan-Meier survival function plots for cessation of RSV shedding stratified by various markers of concurrent RSV infections within the household (HH). Graphs (a) and (b) show results by the proportion of household members infected; graph (c) by the number of persons infected and graph (d) by the presence of another person infected in the household. The legend for each graph is shown in the respective inserted text box.
